# How I Treat: Chronic granulomatous disease

**DOI:** 10.70962/jhi.20250162

**Published:** 2026-04-16

**Authors:** Jennifer W. Leiding, Leah H. Pettiford, Christopher C. Chang, Aimee M. Dassner, Mary C. Dinauer, Benjamin R. Hanisch, Nada Harik, Elizabeth D. Hicks, Harry L. Malech, Felicia B. Morton, Peter E. Newburger, Kathleen E. Sullivan, Brant R. Ward, Michael D. Keller

**Affiliations:** 1 Division of Allergy & Immunology, Johns Hopkins University, Baltimore, MD, USA; 2 Cancer and Blood Disorders Institute and Institute for Clinical and Translational Research, Johns Hopkins All Children’s Hospital, St. Petersburg, FL, USA; 3 Division of Allergy and Immunology, Children’s National Hospital, Washington, DC, USA; 4 Division of Immunology, Allergy and Pediatric Rheumatology, Joe DiMaggio Children’s Hospital, Memorial Healthcare System, Hollywood, FL, USA; 5 Division of Infectious Diseases, Children’s National Hospital, Washington, DC, USA; 6 Division of Pediatric Hematology and Oncology, Washington University School of Medicine, St. Louis, MO, USA; 7 Division of Blood and Marrow Transplantation, Children’s National Hospital, Washington, DC, USA; 8 Laboratory of Clinical Immunology and Microbiology, National Institute of Allergy and Infectious Diseases, Bethesda, MD, USA; 9 CGD Association of America, West Palm Beach, FL, USA; 10 Division of Pediatric Hematology-Oncology, UMass Chan Medical School, Worcester, MA, USA; 11 Division of Allergy and Immunology, Children’s Hospital of Philadelphia, Philadelphia, PA, USA; 12 Center for Cancer and Immunology Research, Children’s National Hospital, Washington, DC, USA; 13 GW Cancer Center, George Washington University School of Medicine, Washington, DC, USA

## Abstract

Chronic granulomatous disease (CGD) is a rare inborn error of immunity with both high risk of invasive bacterial and fungal infections as well as inflammatory complications. Though diagnostic testing via the dihydrorhodamine assay is widely available, disease recognition can be challenging due to the broad range of initial clinical presentations. Preventative antimicrobial therapy is the backbone of management, while treatment of inflammatory disease remains a challenge. Definitive therapy via hematopoietic stem cell transplantation is increasingly favored for resolution of long-term disease risks, while gene therapy remains a promising but investigational treatment. Here, we present our consensus approach to diagnosis and management of CGD.

## Teaser

The authors present their practical guide to diagnosis and treatment of chronic granulomatous disease, including the first comprehensive clinical protocol for treatment of CGD.

## Graphical Abstract

**Figure:**
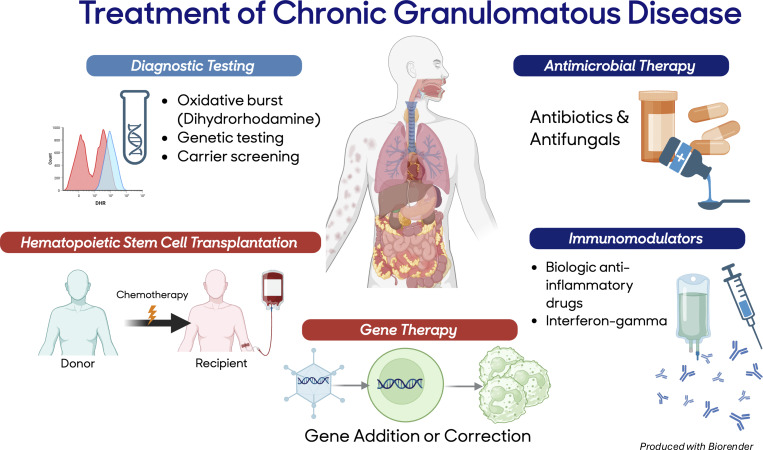

## Introduction

Chronic granulomatous disease (CGD) is a rare inborn error of immunity (IEI) caused by defects in the phagocyte NADPH oxidase complex and is characterized by recurrent and severe invasive bacterial and fungal infections, as well as a high rate of inflammatory disease (1, 2, 3). CGD is caused by defects in six identified genes that make up the NADPH oxidase complex, of which X-linked defects in the *CYBB* gene are most common. Though infections are the most common manifestations, age at presentation and disease spectrum can vary, particularly regarding incidence and severity of inflammatory diseases. Bacteria, including *Staphylococcus aureus*, *Burkholderia cepacia*, *Serratia marcescens*, *Nocardia*, *Klebsiella*, mycobacteria, and fungi, including *Aspergillus* species, represent the most common pathogens in CGD (1, 4, 5, 6). Use of prophylactic therapies, including chronic preventative antimicrobial therapy and interferon γ (IFN-γ), has improved the lifespan of patients, though infections remain a top cause of morbidity and mortality in CGD. Inflammatory disease is also common in CGD, with gastrointestinal disease occurring in roughly half of affected patients (7, 8, 9). Hematopoietic stem cell transplantation has been increasingly recommended for patients with CGD, particularly following the recognition of poorer long-term outcomes in patients with absence of residual phagocyte oxidase activity (10, 11, 12, 13, 14). Use of reduced intensity conditioning and earlier age at transplantation have correlated with improved transplantation outcomes.

Management of CGD is challenging given the rarity of the disease, the phenotypic spectrum, and the limited number of controlled studies in this disease. Here, we gathered expert opinions on the comprehensive management of CGD based on current clinical practice and evidence.

## Functional testing for CGD

**Figure 1.**
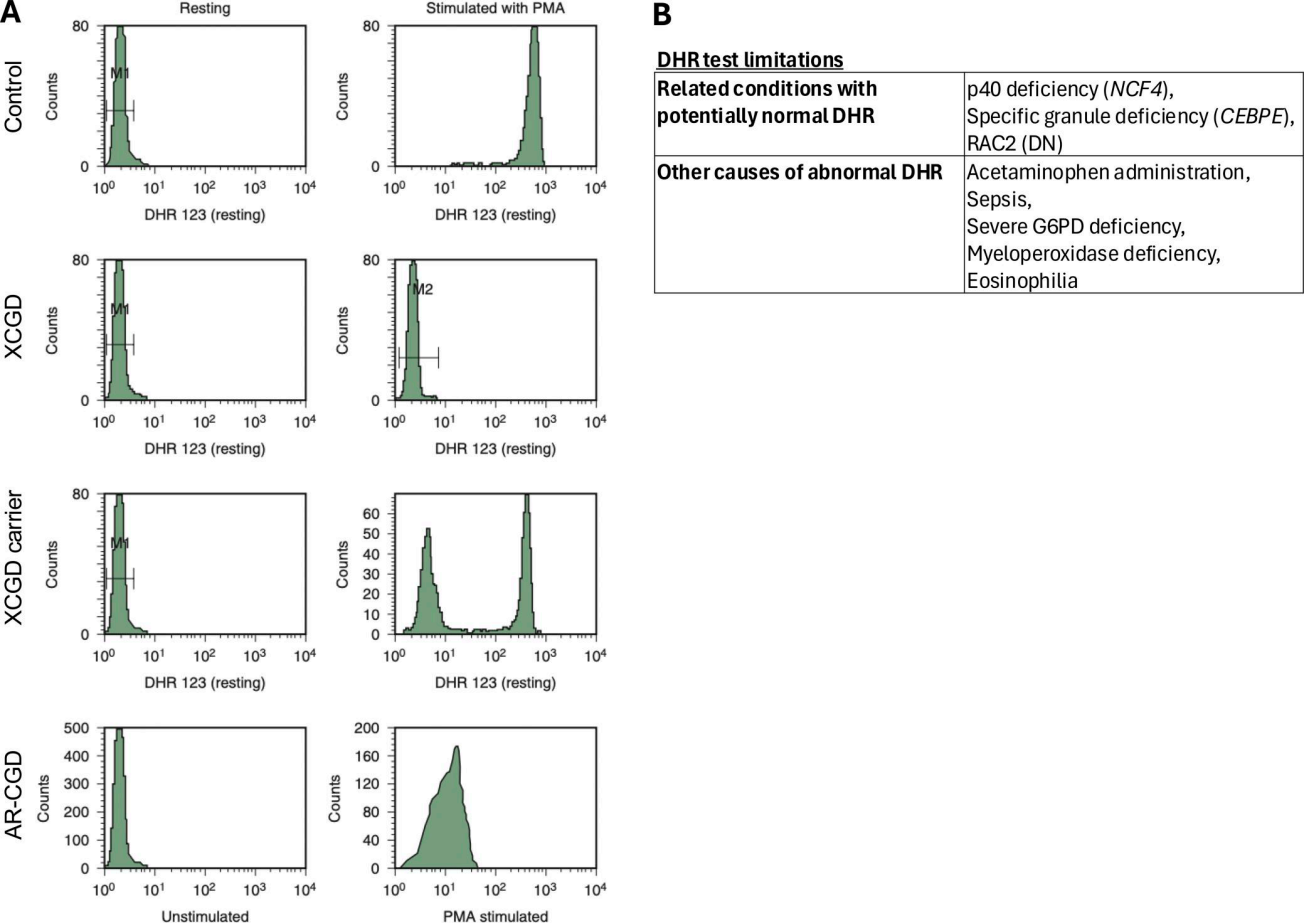
**Use and limitations of the dihydrorhodamine assay for diagnosis of CGD. (A)** Representative histograms from DHR assay (reprinted with permission from *Hematology: Basic Principle and Practice*, Ed. R. Hoffman, eighth edition). The representative autosomal recessive CGD on DHR could also represent hypomorphic X-linked CGD. **(B)** DHR test limitations.

The selection of patients appropriate for an evaluation for CGD is far more difficult than the actual laboratory studies to establish the diagnosis. In practice, there are two tests that are widely available around the world. Both rely on stimulation of neutrophils to activate NADPH oxidase. The nitroblue tetrazolium (NBT) test, which is largely outdated due to its qualitative nature, uses visual scoring of the reduction of NBT to assess superoxide production (15). The current standard for diagnostic testing is the dihydrorhodamine 123 (DHR) assay, a fluorescence-based assay (16), to provide a quantitative assessment of both oxidase activity levels and numbers of active granulocytes. The quantification of DHR reduction correlates directly to NADPH superoxide production and can suggest the possible genotype of CGD. Determination of the proportion of superoxide-producing cells in the DHR assay can also identify female carriers of the X-linked form of CGD, who often have bimodal results. We recommend that in all cases, the histograms generated when performing the DHR be reviewed (Fig. 1 A). Results relying upon DHR-positive gating alone can be misleading, as gating percentages fail to represent quantitative differences in oxidative activity, which are better represented by median fluorescence indices. This is particularly important with respect to recognizing female carriers of X-linked CGD or in those individuals where the *CYBB* gene mutation is a partial function mutation. All patients with the p47phox-deficient autosomal recessive CGD have a right shifted DHR curve similar to partial function mutations of the other CGD genes. Patients with hypomorphic X-linked CGD can also have right shifted DHR curve. Patients with biallelic *NCF4* variants causing a mild atypical form of CGD with a strong inflammatory phenotype are generally not detected by a standard DHR assay using phorbol myristate acetate (PMA) as the stimulus (17). In addition, patients with dominant negative variants in *RAC2* may have abnormal DHR with formyl-methionyl-leucyl-phenylalanine stimulation but not necessarily PMA stimulation (18). While RAC2 is not part of the NADPH oxidase complex, it is a cytosolic regulator of the complex, and some defects have clinical findings that overlap with CGD. Lastly, *PRKCD* deficiency is an immune dysregulation condition associated with low DHR expression (19).

There are also a few scenarios that may lead to a falsely abnormal DHR assay (Fig. 1 B), including eosinophilia and myeloperoxidase (MPO) deficiency (20). Acetaminophen administration as well as sepsis have also been described to transiently suppress oxidative activity on DHR (21).

## Genetic testing

**Figure 2.**
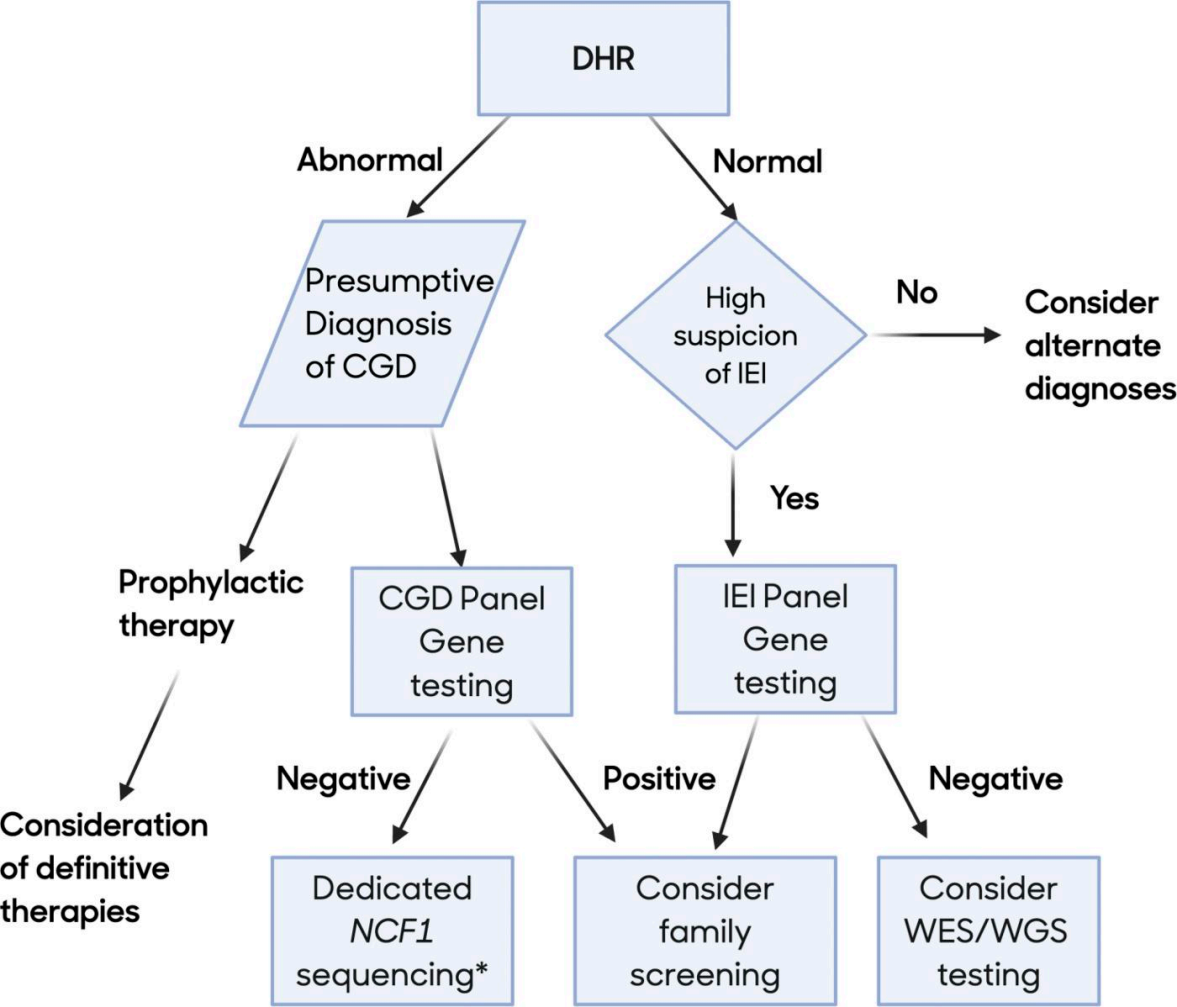
**Diagnostic testing algorithm.** *NCF1 is often not included in many gene panel tests. WES: whole exome sequencing, WGS: whole genome sequencing.

With an ever-increasing number of genes being associated with IEIs, genetic testing has become an integral component in the diagnosis of IEI disorders (22, 23, 24, 25, 26), and we recommend genetic testing in combination with functional testing to help confirm the diagnosis in all suspected cases of CGD (Fig. 2).

One of the primary benefits of genetic diagnosis is to exclude conditions associated with abnormal DHR results, such as MPO deficiency, severe glucose-6-phosphate dehydrogenase deficiency, or Rac2 deficiency, all of which require management that is different from CGD. Knowledge of the specific gene variant may also help to inform decisions regarding definitive treatment, particularly in the potential case of gene therapy (GT). Finally, confirming the genetic cause may help to inform family planning both for the patients and their caregivers. This can be especially important for female carriers of X-linked CGD, who themselves may be at increased risk for infectious and autoimmune complications (27, 28).

The International Union of Immunologic Societies recognizes six genes in which pathogenic variants cause CGD (29). *CYBB* is located on the X chromosome, and the phenotype displays a typical X-linked recessive inheritance pattern. There are also five additional genes—*CYBA*, *CYBC1*, *NCF1*, *NCF2*, and *NCF4*—that display an AR inheritance pattern (2, 3, 30).

For patients with a known familial variant in *CYBA*, *CYBB*, *CYBC1*, *NCF2*, and *NCF4*, we recommend single-gene analysis (e.g., Sanger sequencing) of the gene in question. In patients without a known familial variant, the use of multiple-gene panels is favored to reduce the extra time and expense of repeated single-gene testing. The use of whole-exome or whole-genome sequencing may also be helpful if targeted sequencing has failed to demonstrate a cause for a patient’s symptoms and abnormal DHR testing, including other forms of IEI that have substantial clinical overlap with CGD.

*NCF1* is flanked on both sides by pseudogenes (*NCF1B* and *NCF1C*) with >99% sequence identity, differing from the expressed gene by only a GT dinucleotide deletion (ΔGT) at the beginning of exon 2. The presence of these pseudogenes can make sequencing by both next-generation and Sanger methods challenging (31). As a result, many CGD gene panels exclude *NCF1* or only include analysis of a portion of exon 2. The majority of pathogenic variants in *NCF1* involve a translocation of ΔGT and can thus be identified by targeted analysis of exon 2, though other variants may be missed. Long-read genomic sequencing may be able to effectively differentiate between *NCF1* and its pseudogenes (31). Recently, Hsu et al. also described a bioinformatics approach to effectively identify ΔGT and non-ΔGT variants in NCF1 using existing next-generation sequencing data, which could drastically simplify the identification of *NCF1* variants in the future (32).

## Prophylactic antimicrobial therapy

The most common infections in individuals with CGD vary with geography, though they primarily include catalase-positive organisms, including bacteria such as *S. aureus*, *B. cepacia*, *S. marcescens*, *Salmonella* spp., *Klebsiella* spp, *Nocardia* spp., and *Mycobacterium* spp. and molds such as *Aspergillus* spp. (Table 1) (1, 4).

**Table 1.** Common infections in individuals with CGD

Site of infection	CGD-associated pathogen
Pneumonia	*Aspergillus* species^a^ *Nocardia* species^a^ *Paecilomyces* species^a^ *Phaeohyphomycete* species^a^ *Penicillium* species^a^ *S. marcescens*^a^ *Actinomyces* species *B. cepacia* complex *Klebsiella* species *Mycobacterium* species *Pseudomonas* species *S. aureus*
Lymphadenitis	*Aspergillus* species^a^ *Granulibacter bethesdensis*^a^ *Nocardia* species^a^ *S. marcescens*^a^ *Actinomyces species* *Candida* species *Escherichia coli* *Klebsiella* species *Mycobacterium* species *S. aureus*
Liver abscess	*Aspergillus* species^a^ *Nocardia* species^a^ *S. marcescens*^a^ *S. aureus*^a^ *Actinomyces* species *Candida* species *Streptococcus* species
Brain abscess	*Aspergillus* species^a^ *Nocardia* species^a^ *S. aureus* *Salmonella* species *Exophiala* species
Lung abscess	*Aspergillus* species^a^ *Nocardia* species^a^ *S. aureus* *Actinomyces* species *B. cepacia* complex
Perirectal abscess	*S. aureus* *Klebsiella* species *E. coli*
Skin/soft tissue infection and abscess	*Aspergillus* species^a^ *Chromobacterium violaceum*^a^ *Paecilomyces* species^a^ *Phaeohyphomycete* species^a^ *Penicillium* species *Candida* species *Klebsiella* species *S. marcescens* *S. aureus*
Osteomyelitis	*S. marcescens* ^a^ *Paecilomyces* species^a^ *Aspergillus* species *B. cepacia* complex *Staphylococcus aureus* *M. tuberculosis* *Nocardia* species
Bacteremia/fungemia/sepsis	*B. cepacia* complex^a^ *C. violaceum*^a^ *G. bethesdensis*^a^ *Klebsiella* species *Pseudomonas* species *Salmonella* species *Staphylococcus* species
Meningitis	*B. cepacia* complex^a^ *G. bethesdensis*^a^ *Candida* species *Haemophilus influenzae* *Klebsiella* species

a Pathogens that are pathognomonic for CGD.

Choices for antimicrobial prophylaxis should have activity against the most common and dangerous pathogens, be well tolerated, and have a feasible route of administration and dosing schedule (Table 2).

**Table 2.** Prophylactic therapies for CGD

Category	Medication	Suggested dosing	Potential adverse events	Notes
Antibiotics	Trimethoprim/sulfamethoxazole	5 mg/kg/day TMP, Max 160 mg TMP daily	Hematologic: agranulocytosis, hemolysis, and thrombocytopenia Renal: interstitial nephritis and hyperkalemia GI: abdominal pain, diarrhea, and pancreatitis Dermatologic: photosensitivity and Stevens-Johnson syndrome	Monitor CBC, Cr, and K at baseline and 1 mo Assess G6PD status given potential risk
	**Second line:**	​	​	​
	Levofloxacin	10 mg/kg/dose BID, max 750 mg/dose	MSK: Risk of tendinopathy CV: QTc prolongation Neuro: Peripheral neuropathy and lowering of seizure threshold	Monitor CBC, hepatic panel, and Cr at baseline and 1 mo
Antifungals	Itraconazole	5 mg/kg/day	GI: transaminitis; abdominal pain and diarrhea (in oral suspension) CV: hepatotoxicity (black box warning for ventricular dysfunction) Neuro: peripheral neuropathy Drug–drug interactions due to CYP3A4 inhibitors	Goal drug trough level: ≥1 mcg/ml (incl. metabolites); monitor trough after 7–14 days (if no loading dose given) of dose changes or initiation of any interacting medications, and at 1–3 mo intervals Monitor hepatic panel at baseline and q6 mo Oral formulation and pills are not interchangeable
	Posaconazole	DR tablet:	GI: Transaminitis, potent CYP3A4 inhibitor CV: QTc prolongation	Tablets and suspension formulations are not interchangeable (see Data S1). Goal drug trough level: ≥0.7 mcg/ml
	​	10 to <20 kg: 100 mg BID × 2 doses, then 100 mg daily		
	​	20 to <40 kg: 200 mg BID × 2 doses, then 200 mg daily		
	​	≥40 kg: 300 mg BID × 2 doses, then 300 mg daily		
	Voriconazole	9 mg/kg BID, max 200 mg/dose	GI: transaminitis Neuro: visual disturbance, optic neuritis, vision color changes, hallucination, and peripheral neuropathy CV: QTc prolongation Derm: photosensitivity/dermatologic cancer	Risk of photosensitivity and skin cancers Goal drug trough level: 0.5–5 mcg/ml
Immunomodulators	IFN-γ-1b	50 mcg/m^2^ BSA, or 1.5 mcg/kg/dose (<0.5 m^2^) three times weekly	Fevers, cytopenias, and transaminitis	May induce low-grade fevers with initial medication start, which may be treated or prevented with NSAIDs

G6PD, glucose-6-phosphate dehydrogenase. Cr: creatinine, K: potassium; Gl: glucose; BID: twice daily; q6: every 6 hours; CV: cardiovascular; QTc: corrected QT interval; DR: delayed release

The primary antibacterial regimen utilized for prophylaxis is trimethoprim-sulfamethoxazole (TMP-SMX), which has broad coverage against most bacterial pathogens of concern and is available in oral dosage formulations (33). Implementation of prophylaxis with TMP-SMX has dramatically decreased bacterial infection rates in patients with CGD, with one study reporting reduced incidence of non-fungal infections in AR-CGD from 7.1 to 2.4 per 100 patient months (34). Weight-based TMP-SMX dosing is based on the TMP component, usually at a dose of 5 mg/kg up to a maximum of 160 mg (one double strength tablet) daily. Of note, less frequent dosing of TMP-SMX (akin to those utilized for prevention of *Pneumocystis jirovecii*) is insufficient and not recommended.

There are little data available regarding antibacterial prophylaxis in patients unable to tolerate TMP-SMX prophylaxis, as each other available agent provide less reliable coverage for typical bacterial pathogens of concern. Alternatives include cefpodoxime or levofloxacin (35). A fluoroquinolone can be considered as an option for prophylaxis (33), though are less desirable due to development of bacterial resistance and long-term toxicity risks. Therefore, referral for allergy testing may be beneficial to clarify if a true allergy exists to TMP-SMX and if desensitization is feasible.

Nonbacterial infections are usually fungal—most frequently *Aspergillus *spp.—and carry a higher mortality rate than bacterial infections (4). Itraconazole is the azole antifungal of choice for prophylaxis in this patient population (36); however, notable gaps in itraconazole spectrum of activity include *Aspergillus nidulans*, *Mucormycosis *spp., and some other rare molds. Itraconazole is available in capsules and oral suspension, but these dosage formulations are not interchangeable. The oral suspension is generally preferred due to its enhanced absorption but can cause diarrhea and abdominal pain. When side effects are present, capsules may be preferred. Additionally, the novel super-bioavailable itraconazole dosage formulation overcomes absorption limitations by employing a polymer matrix to enhance enteral bioavailability (37). Itraconazole is both a major substrate and potent inhibitor of CYP3A4; drug–drug interactions with concurrent medications need to be considered.

Given the variability in pharmacokinetics with dosage formulations and absorption, itraconazole therapeutic drug monitoring (TDM) should be considered for CGD prophylaxis, with a suggested goal trough of ≥0.5 mcg/ml (38). Of note, itraconazole TDM measures both the parent drug and its active metabolite; the sum of both should be utilized to assess target concentrations. Achieving target trough levels can be challenging in pediatrics, with one center finding that 70% of children <12 years old were subtherapeutic (<0.5 mg/L) with standard dosing (39).

Despite limited data in the CGD patient population, voriconazole and posaconazole have been utilized for prophylaxis and treatment in CGD. Both have associated toxicities and variability in achieving goal serum concentrations. The phototoxicity with increased risk of skin cancer associated with voriconazole makes it a less appealing option for long-term therapy (40). Therefore, posaconazole is the preferred agent in those unable to tolerate itraconazole or with breakthrough fungal infections. Posaconazole has activity against some dematiaceous molds that are resistant to voriconazole. Prophylaxis with posaconazole should be considered in patients with a previous dematiaceous mold infection. Reaching therapeutic levels with posaconazole can be challenging, particularly when using the immediate release oral suspension dosage formulation. Since the development of posaconazole delayed release tablets—which can be crushed for patients unable to swallow tablets—there has been increased success in achieving adequate serum levels of ≥0.7 mcg/ml for prophylaxis (41, 42, 43, 44, 45). Delayed release suspension is also available for younger children. Patients receiving either voriconazole or posaconazole should be evaluated for associated hepatotoxicity while on therapy via periodic monitoring of liver function tests. Additionally, drug–drug interactions are of concern with both voriconazole (potent inhibitor and substrate of CYP2C9, CYP2C19, and CYP3A4) and posaconazole (potent CYP3A4 inhibitor). Finally, isavuconazole is a newer azole that may have a role in patients where other antifungals may not be appropriate. Data in pediatrics and in CGD are limited.

## IFN-γ therapy

Recombinant human IFN-γ was developed as a prophylactic treatment for CGD based on in vitro studies wherein phagocytes from patients with partial function *CYBB* mutations demonstrated increased superoxide production after treatment with IFN-γ (46). This led to a randomized, double-blind, placebo-controlled study involving 128 CGD subjects in 1991, which showed a significant increase in the time to first infection and a significant decrease in overall serious infections in those receiving IFN-γ versus placebo (47). Based on these results, the U.S. Food and Drug Administration approved recombinant human IFN-γ, administered subcutaneously three times per week, for reducing the frequency and severity of infections associated with CGD.

The mechanism of how IFN-γ treatment leads to decreased infections in CGD patients remains incompletely understood. Additional studies showed that IFN-γ did not enhance NADPH oxidase activity in most patients (48). Later studies hinted at increased nitric oxide production with IFN-γ treatment (49). Recent reports have explored the role of specific NAPDH oxidase alleles and of neutrophil gene expression and metabolic reprogramming (50, 51).

IFN-γ has been safe in prior studies, with no serious adverse events and no discernable effects on growth (52, 53, 54). However, side effects are common, especially fever, myalgias, fatigue, headache, and rash. These symptoms are typically mild and may improve with acetaminophen, non-steroidal anti-inflammatory drugs (NSAIDs), or time. Still, multiple patients in published cohorts required treatment interruption or discontinuation of IFN-γ due to adverse effects.

The pivotal placebo-control trial and many long-term follow-up studies were conducted before the widespread adoption of antifungal prophylaxis for CGD, and data comparing IFN-γ to prophylactic regimens containing azole antifungals are lacking. Small studies have failed to identify differences in rates of infections in patients receiving IFN-γ therapy versus antimicrobial prophylaxis alone (53, 54). A recent meta-analysis supported the use of IFN-γ to prevent infections in CGD, though the authors noted that the 1991 study (before antifungal prophylaxis) carried 80% of the weight of the analysis (55). Thus, the benefit of adding IFN-γ to a prophylactic regimen containing an azole antifungal is still uncertain. Of note, there is no evidence to support use of IFN-γ therapy for the treatment of acute infections.

Given the questions regarding the benefit from adding IFN-γ to a prophylactic regimen of antibiotics and antifungal medications, the authors are in agreement that IFN-γ therapy should be considered on a case-by-case basis with a patient-centered discussion of the risks, benefits, and costs of IFN-γ treatment. Further, use of IFN-γ should not supersede compliant use of antibiotic and antifungal prophylaxis.

## Evaluation and management of infections in individuals with CGD

Infection is often the first clue suggesting the diagnosis of CGD. Common infections in individuals with CGD include pneumonia, visceral abscesses, skin and soft tissue abscesses, lymphadenitis, and osteomyelitis (1). In North America, most infections in patients with CGD are caused by the following organisms: *S. aureus*, *Aspergillus* species*, B. cepacia* complex, *S. marcescens*, *Nocardia* species, and *Klebsiella* species (1, 4, 5, 56). Globally important causes of infection include *Salmonella* species, *Bacille Calmette Guerin* (BCG), and *Mycobacterium tuberculosis* (2). With improvement in molecular pathogen detection, additional organisms have been identified as infectious etiologies. Table 2 details common infections and likely causative organisms (1, 2, 4, 56, 57). In infants, infection with *S. marcescens* is almost pathognomonic of the diagnosis of CGD with bone infections being a common presentation of a *S. marcescens* infection in this age group (4, 58, 59).

When a patient with CGD develops fever or other symptoms concerning for infection, a careful history and physical examination are essential for determining if the clinical picture is consistent with a self-limited infection, such as a viral upper respiratory tract infection that can be managed symptomatically, versus a bacterial or fungal infection requiring laboratory and radiographic evaluation and antimicrobial therapy. Temperature <101^o^F with mild viral symptoms may be monitored at home. Persons with temperature ≥101^o^F for over an hour, or more than one measurement ≥101^o^F over a 24-h period, or prolonged low-grade fever require physician evaluation. Cough, abdominal pain, lymphadenopathy, skin/soft tissue changes, and/or myalgia or musculoskeletal point tenderness require physician evaluation. Clinical symptoms in CGD patients are often subtle even in the presence of a serious infection, and patients may appear relatively well until an infection is very advanced. Determination of the need for outpatient evaluation versus emergency care, therefore, requires consideration of the specific patient’s history, symptoms, and time course.

We suggest the following laboratory studies be obtained for evaluation of possible infection in an individual with CGD: complete blood count (CBC) with differential, C-reactive protein (CRP) or pro-calcitonin, respiratory pathogen PCR panel (if respiratory symptoms are present), liver function tests, aerobic and anaerobic blood cultures, and a methicillin resistant *Staphylococcus aureus* (MRSA) PCR of nares to evaluate for MRSA colonization. Additional laboratory studies should be ordered as indicated by exam and history. To evaluate for a fungal infection, a serum galactomannan or β-d-glucan may be sent. However, sensitivities of these tests are lower in people with CGD than in neutropenic patients, so they have limited diagnostic utility (60). If an infection is not identified on initial workup, we suggest sending plasma microbial cell-free DNA sequencing to aid in diagnosis. Well-appearing individuals with reassuring laboratory results and an identified source of infection may not require hospitalization but should be carefully monitored. Whenever possible based on patient acuity, cultures should be obtained prior to initiation of antimicrobial therapy.

Imaging studies should be dictated by exam and history. Chest computed tomography (CT) should be performed in patients with clinically significant, worsening, or long-standing respiratory symptoms or chest pain. If fevers continue and a source has not been identified, chest, abdominal, and pelvis imaging (CT/magnetic resonance imaging [MRI]) are recommended to evaluate for abscesses. The use of ultralow radiation exposure, no-contrast chest CT is recommended, particularly in the pediatric setting and where multiple follow-up CTs are necessary.

In a patient with lung lesions or consolidations, bronchoalveolar lavage (BAL) should be performed to establish a diagnosis if no pathogen is identified by other testing. BAL fluid should be sent for bacterial, fungal, and acid fast bacillus (AFB) stains and cultures, *Aspergillus* PCR, and galactomannan. Similarly, a tissue biopsy (if anatomically accessible) or drainage procedure should be performed in a patient with infection demonstrated by imaging or exam if no pathogen is identified by other testing. The decision of pursuing biopsy or BAL depends on the location of lung lesions, imaging characteristics, and patient stability. Tissue should be sent for pathology and bacterial, fungal, and AFB stains and cultures. In addition, samples can be sent for molecular genetic testing (broad range PCR and DNA sequencing). If invasive testing cannot be pursued, empiric therapy is reasonable with follow-up imaging, depending on clinical context.

Empiric antimicrobial therapy should account for a patient’s prior infection history, clinical status and acuity, and suspected infection. Initial coverage for a well-appearing patient should cover community-acquired pneumonia and include TMP/SMX and continuing the patient’s current antifungal prophylaxis. Suggested empiric regimen for patients with sepsis is meropenem or cefepime, TMP/SMX, posaconazole or voriconazole, and ± vancomycin (based on the patient’s past infections and MRSA nares PCR result). Suggested empiric regimen for patients with a liver abscess is meropenem or cefepime plus metronidazole, with continuation of the patient’s current antimicrobial prophylaxis. Suggested empiric regimen for patients with lymphadenitis is cefepime plus TMP-SMX or vancomycin (based on past infections and MRSA nares PCR result), with continuation of the patient’s current antifungal prophylaxis. Once a definitive pathogen is identified, antimicrobial(s) should be adjusted to the narrowest regimen that targets the pathogen.

Of note, systemic corticosteroids in addition to antimicrobial therapy have led to improved outcomes in some CGD patients with staphylococcal liver abscesses, severe *Nocardia* pneumonia, and other refractory infections (61, 62, 63). In such cases, we recommend discussion with immunology and infectious diseases specialists to discuss risks and benefits of corticosteroid therapy.

## Inflammatory complications in CGD

**Figure 3.**
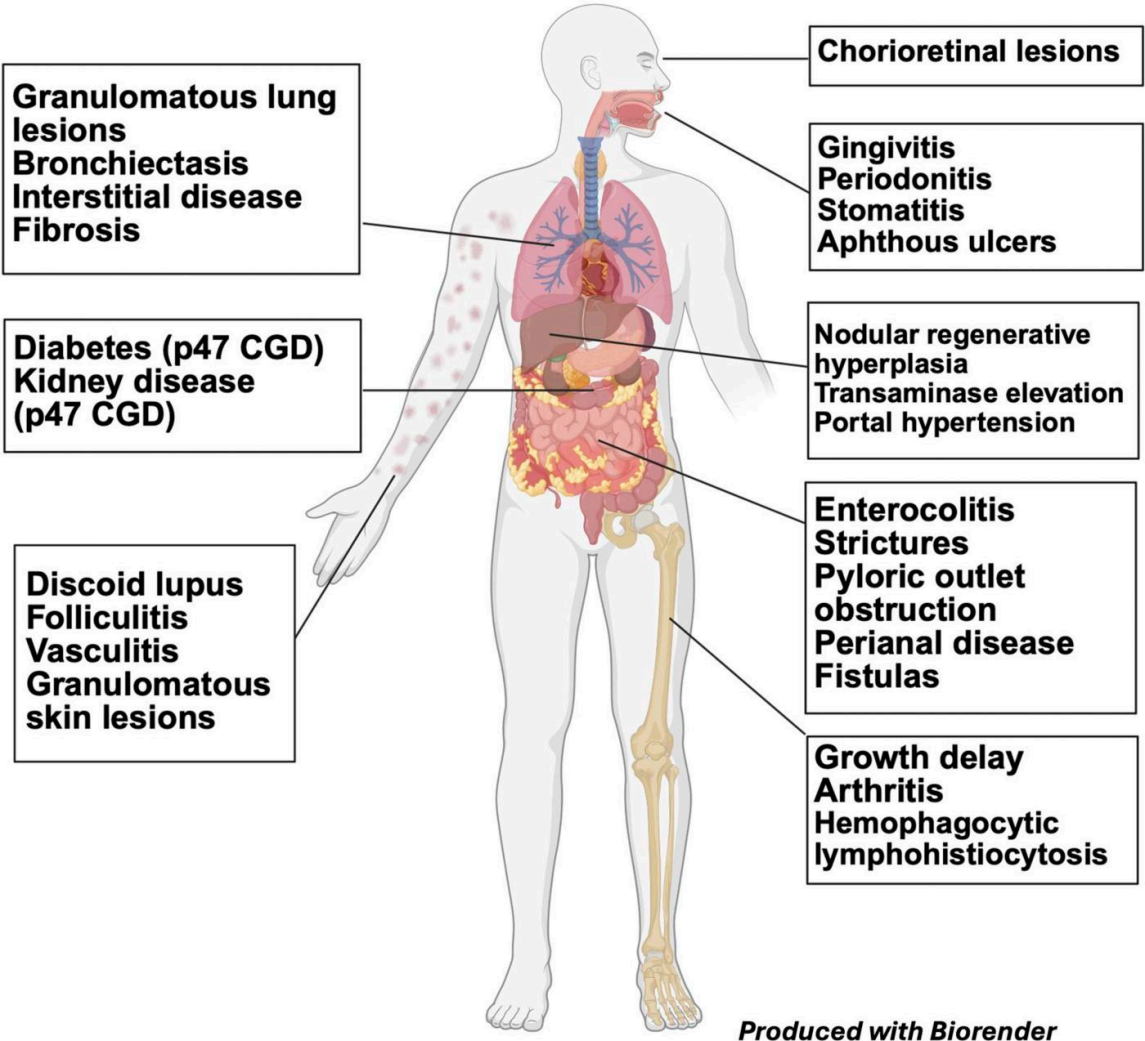
**Inflammatory complications in CGD.** Created in BioRender. Keller, M. (2026) https://BioRender.com/5rfadtd.

Hyperinflammation and autoimmune conditions can be organ specific or can affect multiple organ systems occurring across all genotypes of CGD and are a major source of morbidity (Fig. 3) (7). The gastrointestinal tract, lungs, and liver are the organs most affected. The pathophysiology of hyperinflammation in CGD has not been fully elucidated but has been observed to occur both with and without concurrent infection.

Granuloma formation is a hallmark in CGD and can be sterile or occur at sites of active infection. Granulomas can be obstructive and/or compromise the normal function of the organ that they are infiltrating. Obstructive granuloma at the pylorus can mimic pyloric stenosis in infants and be a presenting sign of CGD (9). Pyloric granulomas with delayed gastric emptying are a common cause of a syndrome of chronic abdominal pain in children with CGD. This may occur in the absence of any other gastrointestinal inflammation. Bladder granulomas can cause pain or difficulty with urination, and long-standing obstructive uropathy can cause chronic urinary tract infections (64). Granulomas respond quickly to corticosteroids, which are the mainstay of treatment, alongside antimicrobial therapy for any active infections. Response to steroid therapy is often diagnostic, which can eliminate the need for imaging or other procedures.

Gastrointestinal inflammation has been reported in up to 50% of patients with CGD in some series and includes manifestations such as esophagitis, protein-losing enteropathy, and inflammatory bowel disease (IBD) (9, 65). Many patients present with classic IBD symptoms that include fever, abdominal pain, weight loss, poor appetite, constipation, diarrhea, melena, and hematochezia. Serum and stool inflammatory markers are often elevated. Clinical presentation often overlaps Crohn’s disease with involvement of the rectum and large intestine with presence of strictures and fistulae. Both lower and upper endoscopic exams are essential for staging the severity and extent of gastrointestinal inflammation and can be supplemented with capsule camera evaluation of the small bowel and/or MR enterography. Endoscopy and capsule camera findings include the presence of skip lesions, ulcerations, and granulomatous inflammation, and MRI has been shown to correlate well with colonoscopy results (66, 67, 68). Steroids and nutritional support are effective in treating protein-losing enteropathy, while treatment of CGD-IBD remains more challenging. Best treatment of CGD-IBD is gleaned from case reports and case series, and any treatment must take into consideration the underlying risk of infection in patients with CGD. Corticosteroids, mesalamine, and antimetabolites such as azathioprine are first-line agents to treat CGD-IBD (7). Although most patients are steroid responsive, up to 70% of patients may relapse and require maintenance steroid therapy. TNFα inhibition is the first-line treatment for pediatric IBD without CGD and is effective in treating CGD-IBD (69) but is not recommended due to the significant infection risk associated with TNFα inhibitors in patients with CGD (70, 71). Anakinra, an IL-1 receptor antagonist, has had mixed responses in CGD (72, 73). Vedolizumab, a humanized monoclonal antibody against integrin α4β7 that prevents adhesion of T cells to mucosal cells, is Food and Drug Administration approved for the treatment of IBD (74). Its use in CGD-IBD has had mixed success with improvement of IBD in some but can be complicated by infections and an inability to wean steroids (75, 76). The most success with biologics has been observed with use of ustekinumab, an IL-12/IL-23 antagonist, with several patients reporting clinical remission of CGD-IBD (77, 78). As is the case with non-CGD-IBD, surgery is avoided with concentration on medical management. However, in life-threatening colitis, especially when hemorrhagic, or in cases of refractory disease, partial colectomy or diverting ileostomies may be used (79). CGD-IBD may spare the small intestine but usually involves the entire colon and rectum. For this reason, ileostomy is preferable to partial colectomy or colostomy as colon inflammation and strictures often recur and/or spread to other parts of the colon and rectum, and colostomy sites are prone to develop inflammation-related fistulae. In patients with very severe and recurrent colonic inflammation and obstructive strictures who have sparing of small bowel disease, ileostomy with total colonic resection may provide long-term amelioration of gastrointestinal inflammatory disease.

Treatment of CGD-IBD requires a multidisciplinary approach between immunology, gastroenterology, and bone marrow transplant specialists. Hemoglobin, MCV, iron studies, liver enzymes, fecal calprotectin, CRP, and erythrocyte sedimentaion rate (ESR) should be routinely monitored in patients with CGD. If concern for IBD is raised, endoscopy and colonoscopy should be performed. For mild symptoms and mild inflammation observed on endoscopy, use of mesalamine is recommended. For moderate to severe disease induction treatment with corticosteroids, a long, slow wean is recommended. Should the patient have a recurrence or continued symptoms, use of a biologic should be highly considered. The presence of CGD-IBD, especially if refractory to treatment, should be a consideration in pursuing definitive therapies such as allogeneic hematopoietic stem cell transplant (HCT). Marsh et al. reported outcomes of CGD-IBD in 145 patients with near total resolution of CGD-IBD by 2 years following HCT (13).

The liver is another major site of inflammation in CGD. Chronic transaminase elevation and hepatomegaly are common, as is drug-induced liver injury (80, 81). Patients living with CGD are on chronic life-long medications and therefore are at risk for iatrogenic liver injury. Nodular regenerative hyperplasia thought to result from liver vasculopathy is a major cause of irreversible liver damage. Vasculopathy is thought to originate from recurrent liver abscesses and liver granulomas that change the architecture of the liver vasculature. Progressive liver disease that leads to portal hypertension is associated with mortality (81). We recommend maximizing nonsurgical treatment of liver abscesses and close monitoring of drug levels to avoid chronic liver injury. However, when chronic liver inflammation is diagnosed, HCT should be highly considered.

Inflammation in the lung can include granulomatous inflammation, interstitial lung disease, bronchiectasis, chronic organizing pneumonia, lymphocytic interstitial pneumonia, and pulmonary fibrosis (82, 83). Chronic or recurrent lung infections likely contribute to the development of chronic lung disease in CGD. Routine imaging and pulmonary function tests are recommended to screen for pulmonary inflammation. A tissue biopsy via transbronchial and/or transthoracic approach is necessary to make a diagnosis of pulmonary inflammation. Treatment with steroids is often effective at treating granulomas and reversible features of interstitial lung disease; however, caution should be taken in the setting of concomitant infection, especially with *Aspergillus*. Pulmonary rehabilitation with mucolytics and chest physiotherapy can also help to manage clinical symptoms associated with chronic lung disease.

Inflammatory skin disease in CGD usually presents as granulomatous acne and can be difficult to treat (84, 85). Topical and systemic corticosteroids often help, but most skin disease remains refractory to treatment. Hydroxychloroquine can be an effective steroid-sparing treatment in some CGD patients with non-acne–related skin inflammation. However, when used, periodic eye examinations are necessary to avoid the ophthalmic toxicities of hydroxychloroquine. Severe hidradenitis both in the axilla and other intertriginous areas can affect some CGD patients. While steroids and/or hydroxychloroquine may help, this is a very difficult inflammation to control. In difficult cases, tofacitinib may offer some control of severe hidradenitis in CGD (Malech, H., personal communication). Inflammation of the oral cavity, including gingivitis, periodontitis, stomatitis, and aphthous ulcers, can be painful and chronic. Swish and spit oral anesthetics and steroids can be effective symptomatic treatments (86). Ocular inflammation is not common, but uveitis, scleritis, and keratitis can be vision threatening (87, 88). Vision changes in a patient with CGD should be urgently assessed by an ophthalmologist.

Systemic autoimmune diseases have been increasingly recognized in CGD, including rheumatoid arthritis, systemic lupus erythematosus, and antiphospholipid syndrome (82). Symptoms of autoimmunity should be assessed at each visit and patients referred to rheumatology when symptoms surface.

At times, hyperinflammation can reach a level as to cause hemophagocytic lymphohistiocytosis (HLH) (89, 90). HLH is a severe, systemic, life-threatening inflammatory disorder caused by cytokine storm and may be clinically indistinguishable from sepsis. In CGD, HLH is most often triggered by infection but also can be secondary to inflammatory disease or autoimmunity. Because of the high mortality associated with HLH, diagnosis requires a high index of suspicion, and treatment relies on rapid initiation of immunosuppression along with antimicrobial therapy.

Although there is a paucity of specific agents to treat inflammation and autoimmunity in CGD, HCT has been shown to resolve the underlying inflammatory disorder (11, 12). Presence of an inflammatory disorder should not delay HCT as data indicate it does not affect transplant survival (12). However, patient performance status does impact HCT outcomes, and therefore, it is imperative to gain as much control as possible of the underlying inflammatory disorder to improve the patient’s overall well-being and performance status before proceeding with HCT.

## Non-antimicrobial preventive measures

While many bacterial and fungal pathogens are ubiquitous in the environment, we recommend certain preventive measures to reduce exposure. As *Aspergillus* and other fungi are highly prevalent in decaying plant matter, avoidance of compost, hay, and mulch is strongly recommended. “Mulch pneumonitis” has been described as a fulminant presenting sign in patients with substantial respiratory exposures (91). Patients should avoid areas where large amounts of dust are being generated (e.g., construction areas). Waterborne bacteria are a potential concern for CGD patients, and avoidance of swimming in fresh or brackish water is important. Zoonotic infections from marine life are a potential risk, as fish and shellfish can harbor *Mycobacterium* species. Infections with *Francisella philomiragia* have been tied to shellfish handling (92). Patients with CGD may safely swim in well-chlorinated pools.

Travel precautions are recommended for patients with CGD due to the potential for water-borne pathogens as well as the potential risk for regional endemic diseases. Mycobacterial infections including *M. tuberculosis* are a risk in patients with CGD, as are waterborne illnesses, including *Salmonella typhi*. Bottled or boiled water is recommended for patients with CGD when visiting or residing in endemic regions (93). These risks should be considered and discussed with patients prior to potential travel. Elective travel to high-risk areas should be deferred particularly if HCT is planned in the near future.

## Vaccinations in CGD

Patients with CGD should receive all vaccines recommended by the American Academy of Pediatrics, except for live bacterial vaccines. Routine live viral vaccines, including mumps, measles, rubella, and varicella vaccines are safe and recommended in CGD. The live yellow fever and oral polio vaccines are also safe to give when indicated.

Live-attenuated bacterial vaccines, including BCG and oral typhoid vaccines, should be avoided (94). In countries where BCG vaccine is given universally at birth, BCG disease occurs in 11–72% of CGD patients (95, 96, 97). This includes local, regional, and disseminated BCG infections. Live-attenuated typhoid vaccines should similarly be avoided given CGD patients’ known susceptibility to *Salmonella* infections. For the same reason, the non-live polysaccharide and conjugate typhoid vaccines should be encouraged in endemic areas.

## Definitive therapy for CGD

Definitive therapies for CGD consist of allogeneic HCT and autologous GT. Compared to conservative management, patients treated with HCT have significantly fewer infections, resolution of inflammatory disease, improved growth, and improved quality of life (12, 98, 99). Patients should be referred for definitive therapy early as outcomes are better in patients who are younger and who have sustained fewer complications from their CGD (11, 12, 100).

HCT is often recommended as standard of care for patients with X-linked CGD or with little to no residual oxidase activity, as absent or very reduced oxidase activity predicts poor survival without definitive therapy (10, 101). In patients with residual oxidase activity, there is less consensus on who is most likely to benefit from allogeneic HCT and weighing overall risk of disease versus the inherent risks of HCT. However, HCT has proven effective for patients with AR-CGD as well (12, 14). Definitive therapy should be strongly considered in those with an available fully matched related donor (MRD), those with severe or refractory infections, including otherwise life-threatening fungal infections, and in those with severe or steroid-dependent inflammatory disease (95). Specifically, CGD-related IBD is cured with HCT and does not appear to adversely affect outcomes from HCT (12, 13).

As in other diseases, outcomes with HCT for CGD are best when using a MRD. If an MRD is not available, a matched unrelated donor (MUD) is the next preferred donor type. Overall survival when using a fully matched donor (related or unrelated) now exceeds 90% (102). Compared to MRD and MUD transplants, transplants using alternative donors, including haploidentical donors, mismatched unrelated donors, and umbilical cord donors, have had inferior outcomes (11, 12, 101). However, these outcomes have been improving more recently. Riller et al. recently found acceptable results when using haploidentical related donors using either α/β T cell depletion or posttransplant cyclophosphamide (103).

Reduced intensity conditioning regimens are preferred over myeloablative regimens in HCT for CGD with matched donors (11, 12, 104). Gungor et al. demonstrated excellent results in matched donor transplants using a reduced intensity regimen consisting of busulfan, fludarabine, and serotherapy (102), Morillo-Gutierrez et al. demonstrated similar results with a treosulfan-based regimen (105), and Chiesa et al*.* found that results were similar between busulfan and treosulfan-based reduced intensity regimens (11). Given the risk of allosensitization, we recommend against the use of granulocyte infusions in patients with CGD, as this has the potential to impact success of later HCT (106).

GT for CGD is a promising but experimental approach to definitive treatment. Early studies in animal models showed that gene transfer into hematopoietic progenitors can lead to oxidase-normal neutrophils (107). A phase 1 human trial using retroviral vectors without conditioning or with minimal conditioning yielded low levels of functioning neutrophils (1 in 5,000) for 2–6 mo. A more recent study of lentiviral GT in nine patients with X-linked CGD evaluated the efficacy and stability of functional reconstitution in the progeny of engrafted cells (108). At 12 months, six of seven surviving patients demonstrated stable vector copy numbers and persistence of between 16 and 46% of oxidase-positive neutrophils. Six of the seven surviving patients were able to discontinue antibiotic prophylaxis, suggesting that autologous GT is a viable approach for the treatment of CGD patients (108).

Challenges have included how to achieve durable engraftment and sustainable clinical benefits while avoiding severe adverse effects due to insertional mutagenesis and growth factor activation leading to myeloid malignancy (109). New and improved lentiviral vectors using bioinformatic mapping of enhancer elements of *CYBB* to take advantage of physiological regulation may improve efficacy (110).

In addition to gene addition, gene editing or gene repair methodologies have been developed. Use of clustered regularly interspaced short palindromic repeats (CRISPR)/Cas9 as well as newer modalities including base editors and prime editors have been used effectively to correct mutations in *CYBB* and *NCF1* both in vitro and in animal models (111, 112, 113). An ex vivo prime editing therapy targeting *NCF1* showed over 75% successful correction in patient HSPCS in preclinical models (114), and in a recent phase I study, two patients were treated with autologous GT utilizing prime editors following busulfan conditioning. The majority of neutrophils demonstrated normal oxidase activity by 1 mo after GT, and gastrointestinal inflammatory symptoms improved in both patients (115).

Commercial development of GT is hindered by the high costs of developing these treatments coupled with the rarity of CGD. Long-term effects of gene editing will need to be addressed. Unmet needs include the refining of in vivo delivery, expanding editing platforms, and ensuring cost-effective access.

## Psychosocial support

There are limited case studies that look at the psychological health of patients with CGD. However, patients diagnosed with chronic illness report higher levels of anxiety, depression, fatigue, and decreased physical activity and quality of life (116), and patients with IEIs experience lower health-related quality of life (117). Patients also experience greater depressive symptoms, pain, and fatigue as more time passes since diagnosis (118). Patients and their families who undergo bone marrow transplants also experience distress and posttraumatic stress symptoms (119). Additionally, caregivers of pediatric patients with chronic diseases experience anxiety and depression (120).

Although additional studies are necessary, we routinely recommend involvement of mental health providers for our patients with CGD and their caregivers (121). Additionally, referral to peer support groups (such as through the Immune Deficiency Foundation and CGD Association of America) for patients and families with CGD may be helpful.

## Evaluation and treatment of XCGD carriers

Female carriers of X-linked CGD may experience decades of symptoms without awareness of their carrier status and are often identified only after a male relative is diagnosed with CGD. Symptoms in known carriers are also often unrecognized or underappreciated. Lyonization, the process by which one X chromosome is randomly inactivated during embryonic development in females, can result in variable proportions of NADPH oxidase active and inactive cells among carriers (27, 122). This phenomenon can affect immune responses and potentially contribute to autoimmune conditions. Female carriers may have a spectrum of symptoms during their lifetime, including infections and multi-organ manifestations. Women with <20% normal superoxide-producing neutrophils are susceptible to infection with CGD-specific organisms like *Aspergillus, Salmonella*, and *Nocardia* (27, 28, 123). Autoimmune manifestations are also common in female carriers and can include gastrointestinal, respiratory, dermatologic, musculoskeletal, neurologic, hematological, and rheumatologic symptoms regardless of the X-inactivation distribution. Oral ulcers, photosensitive rashes, and discoid lupus are some of the most common symptoms. Additionally, carriers may experience irritable bowel syndrome, chronic diarrhea, and IBD. Many carriers self-report psychiatric symptoms like anxiety and depression and receive counseling and/or medication. Given the likelihood of reduced oxidative neutrophil function, female X-linked carriers should not be considered as bone marrow donors for affected male relatives.

There are limited case studies available regarding comprehensive treatment for X-linked carriers. Patients with recurrent infections or carriers with <20% superoxide-producing neutrophils, may require antimicrobial or IFN-γ prophylaxis (28, 123, 124). Those who experience gastrointestinal symptoms may need to be treated with systemic steroids, hydroxychloroquine, topical steroids, biologic modifiers, and surgery. Successful HCT has been performed in some female carriers with very low DHR quantities and significant and recurrent infections, colitis, and autoimmunity, leading to resolution of disease (125, 126). Genetic testing and genetic counseling should be offered to any female with a history of recurrent infections, autoimmunity, or other multi-organ manifestations and a male relative with CGD. Lyonization can change over time, so periodic DHR testing is recommended for female carriers.

## Discussion

CGD, a challenging disease to manage, requires a multispecialty team approach. As a rare disease with multisystem manifestations, provider education remains critical for early recognition. Diagnosis has been aided by broad availability of the DHR assay and genetic testing, and further improvements in genomic data pipelines may hasten genetic diagnosis, particularly for *NCF1* and CGD-like IEIs. Prophylactic therapies have reduced the burden of infections in CGD, though this is limited by the difficulties of maintaining lifelong prophylaxis coupled with the potential for microbial resistance. The recognition of residual oxidative activity as a predictor for long-term mortality has shifted the approach to CGD care, with earlier transplantation strongly favored for patients with absence of oxidative activity in the modern era. Understanding of the burden of disease in female carriers of XCGD has also broadened the patient population and supported the need for comprehensive, longitudinal care for XCGD carriers.

Our approach to the diagnosis and care of patients with CGD is summarized in Data S1. This protocol is limited by both the available evidence and by its nature as a work of expert consensus. However, we are hopeful that standardization of care will facilitate further studies to evaluate and improve the care of patients with CGD.

### Online supplemental material

Data S1 shows the authors' approach to the diagnosis and care of patients with CGD.

## Supplementary Material

Data S1 shows the authors' approach to the diagnosis and care of patients with CGD.
