# Supplementary material for: How I Treat: Chronic granulomatous disease
Source: J Hum Immun. 2026 Apr 16;2(3):e20250162. doi: 10.70962/jhi.20250162 (PMC13177424; doi:10.70962/jhi.20250162)
Supplement: Data S1 — shows the authors' approach to the diagnosis and care of patients with CGD. [file jhi_20250162_datas1.docx]

**Appendix 1: WORK-UP AND MANAGEMENT OF PATIENTS**

**WITH CHRONIC GRANULOMATOUS DISEASE**

*Written collaboratively between the CGDAA Medical Advisory Board and the Divisions of Allergy & Immunology and Infectious Diseases at Children’s National Hospital**

*Authors listed at document end

**1. PURPOSE**

This SOP is designed to standardize the work-up and care of patients with chronic granulomatous disease (CGD) prior to definitive therapy (hematopoietic stem cell transplant [HSCT]. It is intended to apply to outpatient and inpatient management under the Allergy/ Immunology, Bone Marrow Transplant (BMT), and hospitalist services.

1. **BACKGROUND**

Chronic granulomatous disease (CGD) represents a severe form of an inherited error of immunity which requires long term prophylactic therapy or definitive therapy (hematopoietic stem cell transplantation) to restore immunity before potentially fatal infections occur. Treatment includes prophylactic antimicrobials, preventative measures, education, and workup for definitive treatment.

CGD is characterized by recurrent, life threatening bacterial and fungal infections and aberrant inflammation including granuloma formation. CGD is caused by defects in the phagocyte nicotinamide adenine dinucleotide phosphate (NADPH) oxidase complex. These genetic defects result in the inability of phagocytes (neutrophils, monocytes, and macrophages) to destroy certain microbes, as well as defective autophagy and risk of inflammatory disease. The diagnosis is made by neutrophil function testing for superoxide production (DHR). The specific molecular defect causing CGD is determined by genotyping.

Early manifestations of CGD include recurrent upper and lower respiratory tract infections, failure to thrive, visceral abscesses, cellulitis, lymphadenitis, or inflammatory diseases which could include early onset inflammatory bowel disease or sterile granulomatous inflammation which can occur in hollow viscera or other tissues.

Sites of infection include lungs, lymph nodes, skin, and liver.

Most infections in patients with CGD living in North America are caused by 5 organisms:^1^

- *Staphylococcus aureus*
- *Burkholderia cepacia*
- *Serratia marcescens*
- *Nocardia* species
- *Aspergillus* species

1. **WORKUP OF INFANTS WITH ABNORMAL OR ABSENT DHR**
   1. **General principles**
      1. Patients suspected of having CGD should initially undergo neutrophil-function testing using the dihydrorhodamine (DHR) assay.
         1. Dual populations can be seen in the setting of female carriers of X-linked CGD, or in patients with somatic mosaicism.
         2. Mothers, sisters, and other female family members of boys with abnormal DHRs should be offered testing to determine if they have evidence of X-linked carrier status.
      2. A low to absent DHR result warrants treatment and confirmatory genetic testing
         1. DHR intensity must be accounted for in interpretation of the assay, as hypomorphic mutations in CGD genes may diminish rather than eliminate DHR fluorescence.
         2. Patients with highly concerning clinical history but normal DHR should be evaluated for related inborn errors of immunity that may phenocopy CGD.
            1. Rare deficiencies in p40^phox^ (*NCF4*) can be associated with mildly decreased or potentially normal DHR fluorescence in response to PMA stimulation.
         3. Other conditions can result in abnormal DHR
            1. Myeloperoxidase Deficiency
            2. Synovitis, acne, pustulosis, hyperostosis, osteitis (SAPHO) syndrome
            3. Acetaminophen administration has been associated with potentially reduced DHR responses, which normalizes by 24 hrs after dosing in most immunocompetent patients.
   2. **Genetic testing for CGD**
      1. Patients with abnormal DHR tests should undergo genetic testing for underlying molecular cause.
      2. Gene panels or broad-based sequencing (whole exome or whole genome) are acceptable initial genetic tests.
         1. Causative genes include CYBB (XL), CYBA, NCF1, NCF2, NCF4, CYBC1 (AR).
      3. Parental testing should be offered to patients with CGD
         1. X-linked female carriers may have clinical manifestations.
         2. Genetic counseling should be offered to parents of CGD patients, regardless of whether they elect to undergo carrier testing.
      4. Genetic diagnosis of NCF1 (p47phox) deficiency is complicated by presence of pseudogenes**.**
         1. Suspected NCF1 deficiency should be tested by targeted sequencing or by cDNA sequencing.
         2. If NCF1 sequencing is unavailable, p47phox expression may be tested via flow cytometry or western blot (Leidos/NIH).
2. **MANAGEMENT OF CHILDREN WITH CONFIRMED CGD**

3-pronged approach: antibacterial agents, antifungal agents, and IFN-y therapy

- 1. **Prophylactic treatments**
     1. Antibiotic Therapy:
        1. Daily Trimethoprim-sulfamethoxazole (TMP-SMX), beginning at 1 month (5mg/kg/day (based on the TMP component); max dose= 160 mg TMP daily),
           1. Potential Toxicities: Hematologic (agranulocytosis, hemolysis, and thrombocytopenia), renal (interstitial nephritis), metabolic (hyperkalemia), gastrointestinal (abdominal pain, diarrhea, and pancreatitis), dermatologic (photosensitivity, Stevens-Johnson syndrome)

In the setting of early-onset infection or high-risk potential exposure to prior to 1 month of age, therapy could be started with monitoring of bilirubin levels.

- - - - 1. Monitor: CBC, potassium, and creatinine at baseline, at 1 month after initiation of therapy, and approximately every 6 months thereafter
        2. **NOTE**: Prophylaxis is designed to target *S. aureus,* Nocardia, and gram- negative bacteria. Therefore, three times weekly dosing of TMP-SMX used for *Pneumocystis* prophylaxis is NOT appropriate for CGD.
      1. Alternative Medications: in the setting of TMP-SMX allergy or other severe adverse reactions.
         1. Levofloxacin
         2. Second- or third- generation cephalosporin (ex: cefpodoxime)
         3. Consider Allergy referral for evaluation of medication reactions.
    1. Antifungal Therapy:
       1. Initial first line: Itraconazole (5 mg/kg oral solution once daily, maximum dose 200 mg; adult dosing: 200mg once daily)
          1. Potential Toxicities: Hepatitis. Drug-Drug interactions due to CYP3A4 inhibitors
          2. Monitor: liver tests every 6 months while on therapy
          3. Therapeutic Drug Monitoring: obtain serum concentration ≥2 weeks after starting therapy

Trough level: should be collected one hour before scheduled dose.

Goal level (itraconazole PLUS hydroxyitraconazole metabolite): ≥ 1 to 10 mcg/mL

- - - - 1. Drug Interactions: Potent CYP3A4 inhibitor
        2. **NOTE**: Oral formulations (solution and capsule) are NOT interchangeable; oral solution has improved absorption.
      1. Second generation azoles: First line for patients with previous aspergillus infections.
         1. Posaconazole:^2-6^

**NOTE**: Oral formulations are NOT interchangeable; delayed release (DR) dosage formulations (suspension or tablet) are highly recommended when available.

**Delayed release (DR) tablet:** approval for use and conventional dosing recommendations are for patients >40 kg; dosing listed for patients < 40 kg is based on available literature. * If patients are unable to swallow DR tablet, consider crushing tablet(s) and diluting in water;^7^ administer within 1 hour. For patients requiring enteral tube administration, consider using OraBlend (5-8 mL per tablet) as the suspending agent (instead of water) to mitigate clogging.

10 to < 20 kg: 100 mg enterally BID x 2 doses, then 100 mg daily

20 to < 40 kg: 200 mg enterally BID x 2 doses, then 200 mg daily

≥ 40 kg: 300 mg enterally BID x 2 doses, then 300 mg daily

**Delayed release suspension**

10 to < 12 kg: 90 mg enterally BID x 2 doses, then 90 mg daily

12 to < 17 kg: 120 mg enterally BID x 2 doses, then 120 mg daily

17 to < 21 kg: 150 mg enterally BID x 2 doses, then 150 mg daily

21 to < 26 kg: 180 mg enterally BID x 2 doses, then 180 mg daily

26 to < 36 kg: 210 mg enterally BID x 2 doses, then 210 mg daily

36 to < 40 kg: 240 mg enterally BID x 2 doses, then 240 mg daily

**Immediate release (IR) suspension**: decreased bioavailability has been well-described in patients on concomitant proton pump inhibitors or H2RAs, and in those with diarrhea.

< 13 years AND < 34 kg: 6 mg/kg enterally TID

≥ 13 years OR ≥ 34 kg: 200 mg enterally TID

Potential Toxicities: transaminitis, QTc prolongation

Therapeutic Drug Monitoring: obtain serum trough concentration ≥1 week after starting therapy. **Goal level: ≥ 0.7 mcg/mL; if level is subtherapeutic, adjust dose as follows:**

Delayed release tablets: increase dose by 25-50%, rounding to nearest 50 mg (half-tablet); re-check a trough in 5-7 days. E.g: 30 kg patient initially on 200 mg PO daily would increase to 250 mg (1.5 tabs) to 300 mg (2 tabs) daily.

Delayed release suspension: increase dose by 25-50%; re-check a trough in 5-7 days.

Immediate release suspension: increase dose by 25-50%; re-check a trough in 5-7 days. If persistently sub-therapeutic, consider increasing dosing frequency (ex: TID to QID) in consultation with pharmacy OR alternative dosage formulations as available.

Posaconazole Drug Interactions:

Potent CYP3A4 inhibitor. Consider drug interactions if patient requires sirolimus, ergot alkaloids, HMG-CoA reductase inhibitors.

If considering delayed release oral suspension Posaconazole, ask family about known or suspected hereditary fructose intolerance

Goals of Treatment: Limit risk of fungal infections by achieving therapeutic levels with minimal side effects.

- - - - 1. Voriconazole.

Dosing:

Infants and Children < 12 years: 9mg/kg PO q 12h

Adolescents 12 to </=14 years and < 50kg: 9 mg/kg (max 350mg) PO q12h

Adolescents 12 to </= 14 years and >50 kg: 200mg PO q12h

Adolescents >/=14 and < 40 kg: 100mg PO q 12h

Adolescents >/- 15 years >/= 40 kg: 200 mg PO q12h

Potential Toxicities: transaminitis, visual disturbance, optic neuritis, vision color changes, hallucination, peripheral neuropathy, QTc prolongation, photosensitivity/dermatologic

Patients / families should be educated regarding risk of photosensitivity and skin cancers. Regular skin screening is necessary on voriconazole.

Therapeutic Drug Monitoring: obtain serum trough concentration 1 week after starting therapy. **Goal level: ≥ 0.5-5 mcg/mL**

If level is **subtherapeutic**, increase dose by 25-50% and re-check a trough in 3-5 days.

If level is **supratherapeutic,** assess for dose-related toxicities (e.g. visual disturbances, hepatotoxicity) and consult with ID pharmacy to discuss holding dose vs. dose decrease

Drug Interactions: Potent CYP3A4 inhibitor; also inhibits CYP2C19 and CYP2C9

- - 1. Subcutaneous interferon-gamma (Actimmune): May be offered as add-on therapy.
       1. 50mcg/m^2^ BSA, or 1.5mcg/kg/dose (<0.5m^2^) three times weekly
       2. No head-to-head studies of second-generation azoles vs IFN-γ
       3. Risk of cytopenias, hepatitis – requires CBC/hepatic panel monitoring
       4. May induce low-grade fevers with initial medication start, which may be treated or prevented with NSAIDs.
       5. IFN-gamma therapy should be considered more strongly as part of long-term management for patients who are not interested in HSCT and in patients with CYBB splice mutations.
          1. Discussion regarding risks/benefits should be undertaken between the family and primary immunologist.
  1. **Workup and treatment of infections in CGD patients**
     1. Fever Management
        1. Persistent temperatures ≥101F require provider evaluation.
           1. Persistence is defined as T ≥101F for over an hour, or more than one measurement ≥101 over a 24-hour period.
        2. Temperatures <101 F with mild viral symptoms may be monitored at home.
     2. Initial workup for fever or possible infection
        1. Labs:
           1. CBC, CRP or pro-calcitonin, LFTs, respiratory pathogen panel (if respiratory symptoms) should be obtained.
           2. Aerobic and anaerobic blood cultures should be obtained.
           3. If there is no viral or bacterial infection found on initial work-up, consider sending plasma microbial cell-free DNA sequencing (such as the Karius test).
           4. Galactomannan or (BD glucan may be sent, but should not be a replacement for pathogen PCR as the sensitivity is lower than in neutropenic patients.^8^
           5. MRSA PCR of nares to evaluate for MRSA colonization
        2. Imaging:
           1. Chest CT should be performed in patients with clinically significant, worsening, or longstanding respiratory symptoms.
           2. Abdominal/pelvic imaging (ultrasound and/or CT and/or MRI) should be considered to evaluate for abscesses, particularly in absence of an identified source.
           3. Additional imaging should be dictated by exam and history
        3. Disposition: Well-appearing patients with CGD who have reassuring labs which do not show inflammation, and with an identified source of infectious illness may not require hospitalization but should be carefully monitored.
     3. Consultations
        1. Pulmonology consultation is recommended for all CGD patients, particularly with respiratory infections.
        2. ID consultation is highly recommended for all CGD patients admitted with suspected infections.
        3. GI consultation is recommended in the setting of GI symptoms and/or poor growth.
     4. Workup for Infections without identified source.
        1. Procedures including BAL or biopsy should be strongly considered in the setting of infection demonstrated by imaging or exam with no identified pathogen on initial lab testing. Biopsy should be first line if anatomically accessible. BAL fluid should be sent for bacterial, fungal, and AFB stains and cultures, *Aspergillus* PCR, and galactomannan. Tissue should be sent for pathology and bacterial, fungal, and AFB stains and cultures.
        2. Pathogen sequencing, which could include broad range sequencing and/or pathogen-specific testing, should be considered for BAL and tissue samples obtained.
     5. Antibiotic coverage for CGD patients with infections
        1. Empiric antibiotics are appropriate for CGD patients with suspected infections
           1. Holding empiric antibiotics or utilizing narrow spectrum agents until cultures are obtained is reasonable for stable patients, if procedures to procure necessary samples can be performed promptly.
        2. Initial coverage will depend on their prior infection history, acuity, and location of infection
           1. Initial coverage for well-appearing patients should cover community-acquired pneumonia and include TMP/SMX and continuing the patient’s current antifungal prophylaxis.
           2. Suggested initial regimen for patients with:

Suspected sepsis/progressive infection:

Cefepime + TMP/SMX + Posaconazole or voriconazole, +/- vancomycin based on past infections and MRSA nares PCR result.

Meropenem in place of cefepime if patient has hx of prior infection that required a carbapenem for treatment or hx of ESBL

Liver abscess:

Cefepime plus metronidazole with continuation of the patient’s current antimicrobial prophylaxis

Meropenem instead of cefepime plus metronidazole if if patient has hx of prior infection that required a carbapenem for treatment or hx of ESBL

Lymphadenitis:

Cefepime + TMP-SMX or vancomycin (based on past infections and MRSA nares PCR result), with continuation of the patient’s current antifungal prophylaxis

Systemic corticosteroids

Discuss with immunology and infectious disease if starting corticosteroids in the setting of liver abscess or pneumonia would be beneficial^.9^

- - 1. Risk of secondary Hemophagocytic lymphohistiocytosis (HLH)
       1. Secondary HLH is a known potential complication of severe infections in patients with CGD
       2. Screening:
          1. In the setting of sepsis or severe illness, early screening for HLH should be considered, which would include ferritin, CXCL9, sIL2R (CD25), triglycerides, and fibrinogen.
       3. Treatment of secondary HLH:
          1. Resolution of secondary HLH is primarily dependent on effective treatment of the underlying infection.
          2. For patients who meet HLH criteria and are on an effective antimicrobial regimen, adjunctive immunomodulatory therapy should be considered, which could include monoclonal blockade of IL1B (anakinra), IFN-γ (emapulumab), and corticosteroids.
          3. For refractory cases, Janus kinase inhibitors should be considered.
  1. Workup of inflammatory disease in CGD patients
     1. Colitis similar to Crohn’s disease is extremely common in CGD, occurring in >40% of patients
        1. Patients with diarrhea, melena, hematochezia, poor weight gain, or perianal disease should undergo gastrointestinal evaluation.
        2. Stool calprotectin may be used as an initial screen.
        3. Endoscopy and colonoscopy is appropriate as disease extent is often underestimated.
        4. Imaging of the abdomen with CT with contrast may also show signs of colitis.
     2. Patients with obstructive renal or GI disease should be evaluated for granulomatous disease.
        1. If consistent, corticosteroids may be used as initial therapy.
        2. Biologic therapies including IL-23, IL-12/23 and α4β7 antagonists (vedolizumab) should be considered early but require careful initial and longitudinal screening for subclinical infections.
           1. TNFa blockers should be avoided due to risk of severe infections in CGD patients.^10, 11^
     3. Unilateral vision loss should be emergently evaluated by ophthalmology
        1. If consistent with retinal granuloma, oral and/or ocular corticosteroids may be used.
     4. All patients with inflammatory disease who require systemic corticosteroids or biologic therapies should undergo extensive evaluation for subacute infection prior to treatment with immunosuppressive agents
        1. Chest CT should be performed if not done in prior 6 months.
        2. Further evaluations (CT chest/abdomen/pelvis, PET-CT, or other studies) should be considered based on a patient’s history, exam, and symptoms.
  2. Referral and workup for definitive therapy in patients with CGD
     1. All patients with low to absent oxidase activity on DHR should be referred for BMT consultation.
        1. Low to absent DHR activity is highly indicative of poor long-term outcomes in CGD patients who are medically managed without HSCT.
        2. If applicable, clinical trials of gene therapy should be reviewed with the patient and family to determine eligibility and to weigh risks/benefits of gene therapy versus HSCT.
     2. Patients with CGD should undergo HLA testing, as should all full biologic siblings and parents.
        1. Sisters and mothers of boys with XCGD should undergo testing for carrier status, as carriers would not be suitable donors for HSCT.
  3. **Vaccination**
     1. Patients with CGD should receive all routine childhood vaccinations given in North America.
     2. COVID-19 vaccinations are safe and recommended in CGD
     3. Live bacterial vaccines are contraindicated in patients with CGD
        1. Live bacterial vaccines include:
           1. Oral typhoid vaccine (given for travel to typhoid endemic regions)
           2. Bacillus Calmette-Guerin (BCG) vaccine (not utilized in US)
  4. **Family Vaccinations**
     1. Families of an infant with CGD should receive all appropriate vaccines to secondarily protect the immunocompromised patient. Particular focus should be given to:
        1. Seasonal inactivated influenza vaccine
        2. COVID-19 vaccinations and boosters
        3. DTaP (all should have received within the previous 5 years due to risk of pertussis in school aged children)
     2. Live vaccines **may** be given to family members of CGD patients
        1. MMR and varicella may be given to siblings or extended family. If rash develops at the site of varicella vaccine (Varivax or zostavax), the rash should be kept covered, and the individual kept away from the immunocompromised patients.
        2. For family members being evaluated as bone marrow transplant donors, speak with BMT team prior to live vaccinations.
  5. **Protective Measures in the Home**
     1. Preventative measures
        1. No swimming in fresh or brackish water (rivers, lakes, estuaries)
           1. Swimming in chlorinated pools is safe.
        2. Avoid construction sites or areas where a large amount of dust is generated. Patients should mask if this cannot be avoided
        3. Avoid farm exposures as well as decaying plant matter (mulch, hay, compost). Patients should mask if this cannot be avoided
        4. Avoid mowing the lawn or gardening or being in proximity of lawn mowing.
        5. Drinking water precautions:
           1. Public tap water and bottled water is safe for CGD patients
           2. Water from any other sources should be boiled prior to drinking.
        6. Discuss with health care team prior to travel regarding risk of endemic infections.
           1. Fungal infections (phaeohyphomycosis in tropical and subtropical areas)
           2. Typhoid and mycobacterial infections (international travel to tropical and developing nations).
     2. Pediatricians should remain involved in the care of CGD patients for routine vaccinations and preventative care.
     3. Sick caregivers are encouraged to avoid contact with the patient if possible.
        1. Though routine viruses do not pose a risk to CGD patients, any high fevers and respiratory illnesses may require evaluation to rule out bacterial/fungal infections.
        2. In the event that there are no alternative caregivers, the caregiver should use a mask if respiratory symptoms are present and minimize close contact with the patient.
  6. **Referral for evaluation of known or suspected carriers of X-linked CGD**
     1. Female carriers of X-linked CGD (XCGD) often have clinical manifestations which can include autoimmune disease and infections^12^
     2. Clinical manifestations in female XCGD carriers can vary in severity depending on degree of skewed X chromosome lyonization.
     3. Early referral for immunologic testing and treatment is strongly recommended for all first-degree female relatives of XCGD patients.
  7. **Family Psychosocial Support**
     1. All families should be given contact information for social work and psychology services early after diagnosis.
     2. Psychosocial support for parents and caregivers
        1. Screening for mental health disorders is recommended.
        2. Although therapy may not be available for parents who are experiencing psychosocial distress related to their child’s diagnosis, social work can provide immediate support at the time of appointment which includes financial resources, a list of support groups, and assistance with outside therapy referrals.
        3. Families should be offered the chance to connect with other families who have similar diagnosis through the Immune Deficiency Foundation and CGD Association of America.
     3. Psychosocial support for patients
        1. Psychology involvement should be considered for children over the age of 5 years who are experiencing psychosocial distress related to their diagnosis or a sibling’s diagnosis.
        2. Direct referral to institutional providers should be provided if available, and/or social work involvement should be utilized to identify community providers and resources.
     4. Social worker should meet with family during initial BMT consult and/or during Pre-Transplant workup to discuss logistics and offer financial/emotional supportive services to the family
     5. Informational support / advocacy
        1. Families should be given appropriate chapters from the Immune deficiency foundation patient handbook
        2. Families considering bone marrow transplant should be given the institution-specific information on bone marrow transplantation (if available)
        3. Families should be given information regarding the Immune Deficiency Foundation and CGD Association of America to facilitate involvement in these communities.
  8. **Isolation Procedures in the ER and Hospital settings**
     1. If Emergency room referral is necessary, it is critical that the Emergency department staff know that the patient is immunocompromised and must not be placed in the waiting room, and requires isolation with mask, gown, and gloves
     2. Hospital Admission
        1. Patients with CGD should be strictly isolated from other patients with *Burkholderia* or mycobacterial infections.
           1. Ideally, patients should not have care providers who are concurrently caring for other patients with these infections.

*Authors*:

Leah Pettiford FNP^1^, Brant Ward MD PhD^1^, Elizabeth Hicks MD^1.2^, Naynesh Kamani MD^1^, Suzannne Kochis MD^1^,

Priyanka Seshadri MD^1^, Benjamin Hanisch MD^3^, Aimee Dassner PharmD^3^, Nada Harik MD^3^, Michael D. Keller MD^1^

and the CGDAA advisory board members & leadership

1 Division of Allergy & Immunology, Children’s National Hospital

2. Division of Blood and Marrow Transplantation, Children’s National Hospital

3. Division of Infectious Diseases, Children’s National Hospital

**References**:

1. Thomsen IP, Smith MA, Holland SM, Creech CB. A Comprehensive Approach to the Management of Children and Adults with Chronic Granulomatous Disease. *J Allergy Clin Immunol Pract*. Nov-Dec 2016;4(6):1082-1088. doi:10.1016/j.jaip.2016.03.021

2. Mason MJ, McDaneld PM, Musick WL, Kontoyiannis DP. Serum Levels of Crushed Posaconazole Delayed-Release Tablets. *Antimicrob Agents Chemother*. May 2019;63(5)doi:10.1128/AAC.02688-18

3. Bernardo V, Miles A, Fernandez AJ, Liverman R, Tippett A, Yildirim I. Initial posaconazole dosing to achieve therapeutic serum posaconazole concentrations among children, adolescents, and young adults receiving delayed-release tablet and intravenous posaconazole. *Pediatr Transplant*. Sep 2020;24(6):e13777. doi:10.1111/petr.13777

4. Mauro M, Colombini A, Perruccio K, et al. Posaconazole delayed-release tablets in paediatric haematology-oncology patients. *Mycoses*. Jun 2020;63(6):604-609. doi:10.1111/myc.13084

5. Garner LM, Ngo S, Kaplan JB, Wilson WS, McKinzie CJ. Evaluation of Posaconazole Dosing in Children and Young Adults: A Single-Center Review. *J Pediatr Pharmacol Ther*. 2021;26(8):834-840. doi:10.5863/1551-6776-26.8.834

6. McCann S, Sinha J, Wilson WS, McKinzie CJ, Garner LM, Gonzalez D. Population Pharmacokinetics of Posaconazole in Immune-Compromised Children and Assessment of Target Attainment in Invasive Fungal Disease. *Clin Pharmacokinet*. Jul 2023;62(7):997-1009. doi:10.1007/s40262-023-01254-2

7. Bio LL, Hiroshima L, Schwenk HT, Green S. Successful enteral administration of crushed posaconazole delayed-release tablets in children. *Pediatr Blood Cancer*. Feb 2024;71(2):e30782. doi:10.1002/pbc.30782

8. King J, Henriet SSV, Warris A. Aspergillosis in Chronic Granulomatous Disease. *J Fungi (Basel)*. May 26 2016;2(2)doi:10.3390/jof2020015

9. Leiding JW, Freeman AF, Marciano BE, et al. Corticosteroid therapy for liver abscess in chronic granulomatous disease. *Clin Infect Dis*. Mar 1 2012;54(5):694-700. doi:10.1093/cid/cir896

10. Uzel G, Orange JS, Poliak N, Marciano BE, Heller T, Holland SM. Complications of tumor necrosis factor-alpha blockade in chronic granulomatous disease-related colitis. *Clin Infect Dis*. Dec 15 2010;51(12):1429-34. doi:10.1086/657308

11. Conrad A, Neven B, Mahlaoui N, et al. Infections in Patients with Chronic Granulomatous Disease Treated with Tumor Necrosis Factor Alpha Blockers for Inflammatory Complications. *J Clin Immunol*. Jan 2021;41(1):185-193. doi:10.1007/s10875-020-00901-8

12. Miranda MA, Tsalatsanis A, Trotter JR, et al. High symptom burden in female X-linked chronic granulomatous disease carriers. *Clin Immunol*. Nov 2024;268:110364. doi:10.1016/j.clim.2024.110364
